# Altered Brain Function in Drug-Naïve Major Depressive Disorder Patients With Early-Life Maltreatment: A Resting-State fMRI Study

**DOI:** 10.3389/fpsyt.2019.00255

**Published:** 2019-04-24

**Authors:** Zhexue Xu, Jing Zhang, Di Wang, Ting Wang, Shu Zhang, Xi Ren, Xiaolei Zhu, Atsushi Kamiya, Jiliang Fang, Miao Qu

**Affiliations:** ^1^Department of Neurology, Xuan Wu Hospital Capital Medical University, Beijing, China; ^2^Department of Neurology, Third Affiliated Hospital, Beijing University of Chinese Medicine, Beijing, China; ^3^Department of Clinical Psychology, Beijing Anding Hospital, Beijing, China; ^4^Nanjing Municipal Hospital of Traditional Chinese Medicine, Nanjing, China; ^5^Department of Neurology, Fengtai Integrated Chinese and Western Medicine Hospital of Beijing, Beijing, China; ^6^Department of Radiology, Guang’ anmen Hospital China Academy of Chinese Medical Sciences, Beijing, China; ^7^Department of Psychiatry and Behavioral Sciences, Johns Hopkins University School of Medicine, Baltimore, MD, United States

**Keywords:** major depressive disorder, childhood maltreatment, resting state, fMRI, prefrontal-limbic system

## Abstract

Childhood Maltreatment (CM) is an important risk factor for major depressive disorder (MDD). Previous studies using emotional task-state functional magnetic resonance (task-state fMRI) found that altered brain function in prefrontal-limbic regions was the key neuropathological mechanism in adult MDD patients with experience of early-life maltreatment. However, to the best of our knowledge, there is no published study investigating brain function in MDD patients with CM experience using resting-state fMRI (rs-fMRI). In present study, we aimed to detect altered resting-state brain activity in MDD patients with CM experience, and identify significantly activated brain regions, which may provide new insights into the neural mechanism underlying the relationship between MDD and CM experience. The results showed MDD patients with CM experience were associated with increased amplitude of low-frequency fluctuation (ALFF) and altered function connection (FC) in the prefrontal cortex, when compared to MDD patients without CM. Of note, left frontal middle gyrus (LFEG) was found as a specific brain region which differentiates MDD patients with CM from patients without CM. These results suggest that rs-fMRI is a useful method in studying the correlation between MDD and CM experience and altered function of LFEG in resting-state may explain the correlation between MDD and CM experience.

## Introduction

Major depressive disorder (MDD) has become the single largest contributor to nonfatal health loss globally in 2015 (1). The causality of MDD is heterogeneous. Although stress, poor family relationship and social support in adulthood have been thoroughly studied as environmental risks for MDD, unpleasant psychical or physical experiences during childhood were often overlooked. Childhood maltreatment (CM) has recently gained greater attention because it may confer susceptibility to depression in later-life. Clinical evidence from retrospective and prospective cohort studies suggests that CM could markedly increase the risk of MDD (2–5). Furthermore, it has been reported that approximately 65% of chronically depressed patients have a history of CM, which is associated with more relapses and heightened therapy resistance (6, 7). Therefore, it is important to identify the neural mechanisms underlying the impact of CM on MDD pathophysiology, for pursuing early intervention and mechanism-based treatment strategies.

CM has been proved to affect brain function and development in MDD patients (8–11). Task- and resting-state functional magnetic resonance imaging (fMRI) has been wildly used to non-invasively evaluate functional brain activity for identification of specific brain regions and neural circuits associated with disease conditions. Previous studies using emotional task-state Fmri (12–15) showed altered activation of prefrontal-limbic regions, including ventromedial prefrontal cortex (vmPFC), anterior cingulate cortex (ACC), amygdale and hippocampus, in MDD patients with CM.

Nonetheless, to our best knowledge, resting-state brain function in MDD patients with CM still remains to be investigated. In the present study, we utilized rs-fMRI to examine the neuropathological mechanisms of drug-naïve MDD with CM experience. We hypothesize that altered blood oxygenation level dependency (BOLD) in certain brain regions may be correlated with CM experience in MDD patients and these brain areas may include subregions of the prefrontal-limbic system, which has previously been reported to be associated with MDD patients with CM using task-state fMRI.

## Methods

### Participants

Fifteen MDD patients with CM and fifteen patients without CM were recruited from the outpatient clinic of Xuanwu Hospital Capital Medical University, Third Affiliated Hospital of Beijing University of Chinese Medicine, and Beijing Anding Hospital. All of the patients were diagnosed with modified structure clinical interview for DSM-V (16) by two senior clinical psychiatrists, and were rated with a 17-item Hamilton depression scale (HAMD). All MDD patients were drug-naïve and in their first episode of illness. These patients were right-handed and would be excluded if they had another major psychiatric illness, neurological illness, head injury, alcohol or drug abuse. CM was assessed by a short form childhood trauma questionnaire (CTQ-SF) (17).

Seventeen age-, gender- and education-matched healthy controls (HC) were recruited from community-based advertising through flyers posted at hospital and university campuses. They were also interviewed with the Structured Clinical Interview for DSM-V. All HC were right-handed, free of depression and any other psychiatric or neurological illness and had no history of head injury, alcohol or drug abuse.

### Data Acquisition

T1-weighted and resting-state fMRI data were acquired using a 3T Siemens Trio scanner (Magnetom Allegra, Siemens, Erlangen, Germany) in the Beijing Guang’ anmen Hospital China Academy of Chinese Medical Sciences. The scanning sessions included the following: (i) three-dimensional T1-weighted whole-brain images: 3D-MPRAGE sequence, Repetition Time (TR)/Echo Time (TE) = 2300/3ms, 176 sagittal slices. (ii) Rs-fMRI scans contain 180 functional volumes, using a T2-weigthed Echo Planar Imaging sequence, TR/TE = 2,000/30 ms, flip angle = 90°, acquisition matrix = 64 × 64 axial slices = 40, thickness/gap = 3/0 mm, Voxel size: 3.0 × 3.0 × 3.0mm (3), Field of view = 210 × 210 mm. During the scanning, subjects laid supine in the scanner with their heads fixed with foam pads to decrease head motion. They were informed to close their eyes but remain awake, and a simple inquiry was conducted to exclude any sleeping periods.

### Pre-Processing

Image preprocessing and statistical analysis were performed using the Data Processing Assistant for Resting-state fMRI (DPARSF, http://www.rfmri.org/DPARSF) toolkits (18), Resting State fMRI Data Analysis Toolkit 1.8 version (REST, https://www.nitrc.org/projects/rest/) (19) and SPM8 software (SPM8, http://www.fil.ion.ucl.ac.uk/spm/) (20).

Images were drafted by REST and BrainNet Viewer toolkit. Data pre-processing was performed by DPARSF toolkits. The steps were as follows: (i) Raw DICOM data were converted to the NifTI format; (ii) To allow for instrumental stabilization of the initial signal, first 10 images were discarded; (iii) Images were slice-timing and 3D motion corrected for head motions, we excluded images if patients’ and HC’s head movement data in translational and rotational planes i.e. exceeded 2mm or 2° and 1mm or 1°; (iv) Images were normalized based on the Montreal Neurological Institute (MNI) Space with Smoothing Method (Full Width at Half Maximum, FWHM 4mm); (v) rs-fMRI data were processed with linear detrending and band-pass filtering.

### ALFF and FC Analysis

After pre-processing, very low-frequency drift and high-frequency noise was first filtered (band-pass, 0.01∼0.08Hz), and then a Fast Fourier Transform (FFT) was used to convert the frequency domain. This averaged square root was termed Amplitude of Low-Frequency Fluctuation (ALFF) at the given voxel (21). Furthermore, in order to eliminate the physiological signals, fractional ALFF (fALFF) was also performed. In the following FC analysis, according to our present results and referenced by previous CM task-fMRI study results (22), the left orbital part of inferior frontal gyrus, left anterior cingulated and paracingulate gyri, left middle frontal gyrus and left inferior parietal, extending to supramarginal and angular gyri, were chosen as a region of interest (ROI). After, seed-to-voxel functional connectivity was performed.

### Statistical Analysis

Subjects’ demographic information, including age, gender, education level, and their matched HC groups were analyzed by One-way ANCOVA. Gender related differences were detected by Chi-square tests. ALFF, fALFF and FC results were performed in correlation with CM scale using Pearson Correlation. The statistical significance level was set at *p* < 0.05. All statistical tests were performed using SPSS 18.0 (SPSS Inc., IL, USA).

The technologists who performed fMRI data analysis were blind to the subjects. Significant brain activation in the whole brain was computed using one-sample t-test in REST (Threshold *p* < 0.05) for every group. Voxel-wise group comparisons were detected with two-sample t-test (AlphaSim correction *p* < 0.01; continuous voxels > 16). The precise anatomical position in the brain, with statistical significance on the corresponding MNI coordinate, was identified using the Viewer in REST. Voxel-wise FC analyses revealed the Pearson correlation coefficients between the seeds and the rest of the whole brain areas. Fisher r-to-z transformation were used to transform FC values into z-values. The group differences in the functional connectivity (AlphaSim correction *p* < 0.01; continuous voxels > 16) were disclosed using two sample t-tests.

## Results

### Demographic and Clinical Characteristics of the Study Group

MDD patients (n = 15 MDD with CM and n = 15 MDD without CM) and matched HC (n = 17) participated in this study. As a subject in MDD without CM group was excluded for a big head motion, there were 14 subjects in MDD without CM group in practice. The demographic information, HAMD scores, and CM scores for these groups were shown in **Table 1**. There were no statistical differences in age, gender, and years of education between the groups. The MDD with or without CM showed higher HAMD scores compared to those in matched HC (MDD with CM: 26.33 ± 7.99; MDD without CM: 24.14 ± 4.88, HC: 1.06 ± 1.19, *p* < 0.01), whereas no significant difference in HAMD scores between CM and without CM groups was observed. MDD with CM had a higher CM score than MDD without CM and HC (MDD with CM: 63.33 ± 4.03; MDD without CM: 31.16 ± 5.63, HC: 30.83 ± 4.02, *p* < 0.01), and there was no significant difference found between MDD without CM and HC.

**Table 1:** Demographic and psychological data of MDD with CM, MDD patients without CM and controls.

	MDD with CM	MDD without CM	Control	χ*^2^*/ F	P-value
No. of subjects	15	14	17		
Gender (M/F)	6/9	5/9	7/10	*χ^2^* = 0.10	*p* = 0.95
Age, years (mean, SD)	28.33 ± 5.81	32.36 ± 6.23	28.94 ± 5.92	*F* = 1.90	*p* = 0.16
Education, years (mean, SD)	16.13 ± 3.09	16.14 ± 3.11	16.18 ± 2.81	*F* = 0.001	*p* = 0.99
HAMD score (mean, SD)	26.33 ± 7.99	24.14 ± 4.88	1.06 ± 1.19	*F* = 110.84	*p* < 0.01
CM score (mean, SD)	63.33 ± 4.03	31.16 ± 5.63	30.83 ± 4.02	*F* = 97.71	*p* < 0.01

MDD, Major Depressive Disorder; HAMD, Hamilton Depression Rating Scales; Age, Education and HAMD score adopted one-way ANOVA; CM, Childhood Maltreatment. There is no difference between MDD with CM and MDD without CM in HAMD scores (p = 0.82, Bonferroni corrected). MDD with CM had significant differences with MDD without and Control in CM scores respectively (p < 0.01, Bonferroni corrected), and there is no significant difference between MDD without CM and HC (p = 0.90, Bonferroni corrected).

### ALFF and fALFF Analysis

**Figure 1:**
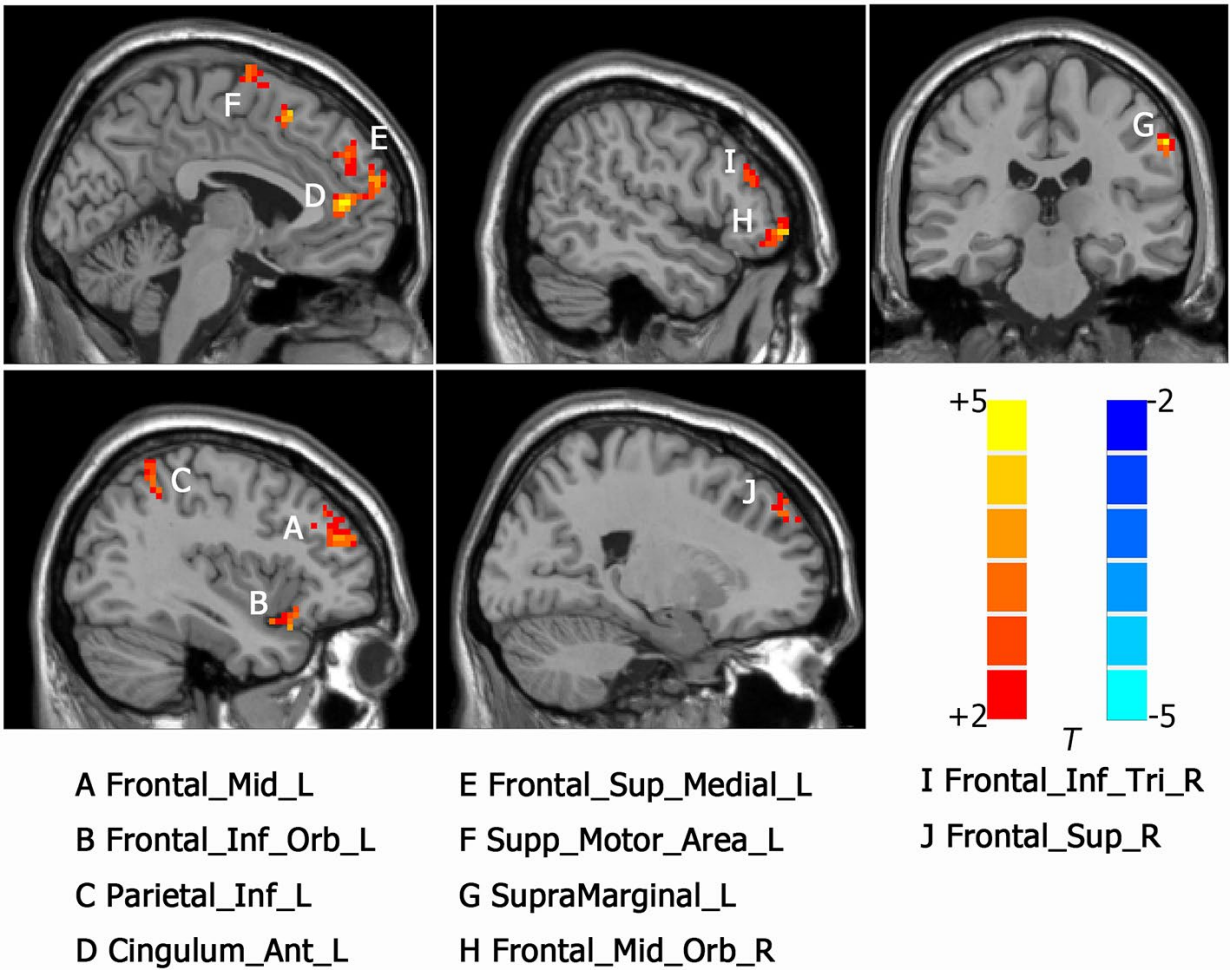
Activated brain regions showed by rs-fMRI using method of Amplitude of Low-Frequency Fluctuation (ALFF) in MDD patients with childhood maltreatment (CM) compared with the health control (HC). A: Frontal_Mid_L, left middle frontal gyrus; B: Frontal_Inf_Orb_L, left inferior frontal gyrus, orbital part; C: Parietal_Inf_L, left inferior parietal, but supramarginal and angular gyri; D: Cingulum_Ant_L, left anterior cingulated and paracingulate gyri; E: Frontal_Sup_Medial_L, left superior frontal gyrus, medial; F: Supp_Motor_Area_L, left supplementary motor area; G: SupraMarginal_L, left supramarginal gyrus; H: Frontal_Mid_Orb_R, right superior frontal gyrus, medial orbital; I: Frontal_Inf_Tri_R, right inferior frontal gyrus, triangular part. J: Frontal_Sup_R, right superior frontal gyrus, dorsolateral.

**Figure 2:**
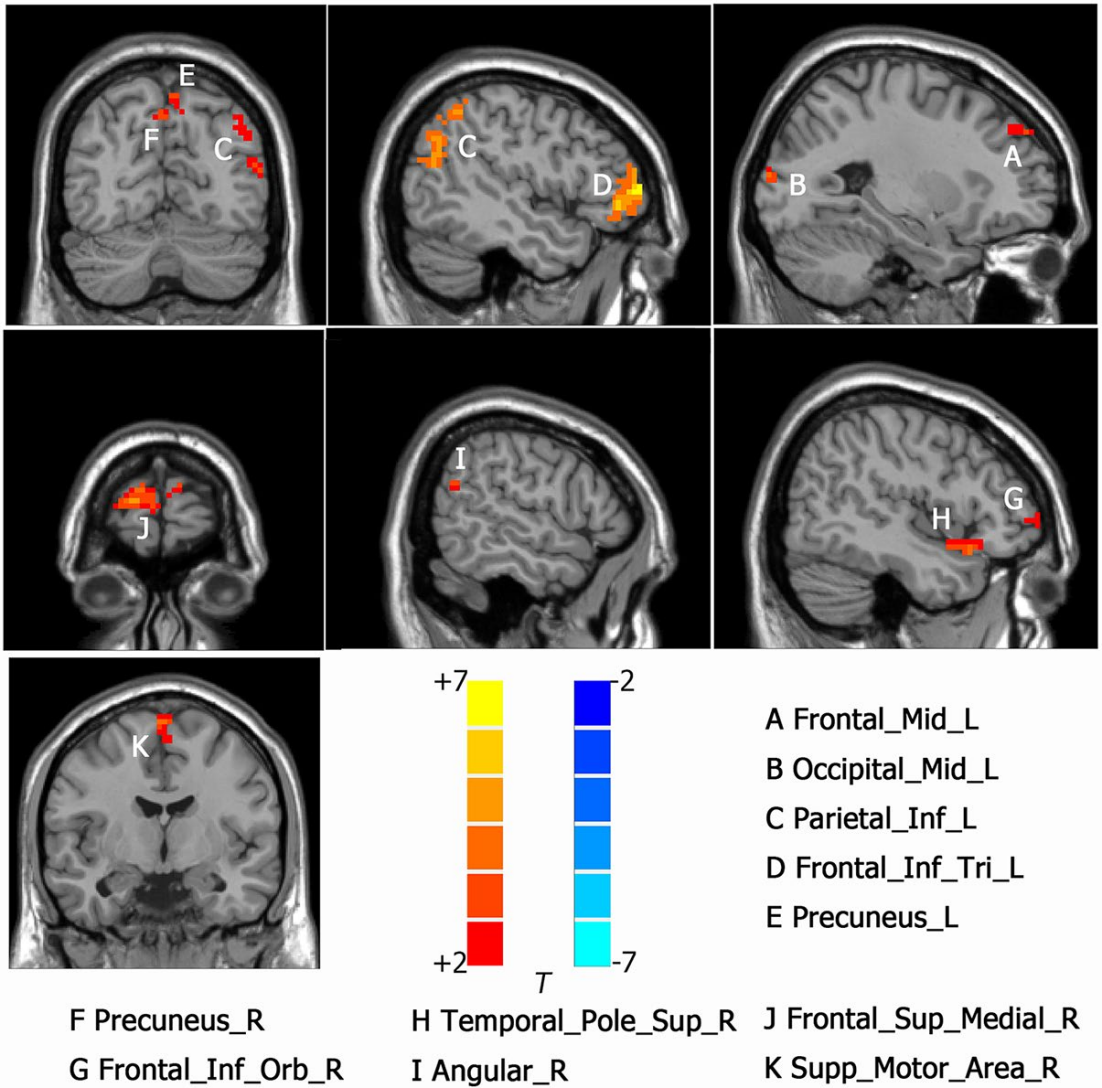
Activated brain regions showed by rs-fMRI using method of ALFF in MDD patients without CM compared with the HC. A: Frontal_Mid_L, left middle frontal gyrus; B: Occ:pital_Mid_L, left middle occipital gyrus; C: Parietal_Inf_L, left inferior parietal, but supramarginal and angular gyri; D: Frontal_Inf_Tri_L; left inferior frontal gyrus, triangular part, E: Precuneus_R, left precuneus; F: Precuneus_R, right precuneus; G: Frontal_Inf_Orb_R, right inferior frontal gyrus, orbital part; H Temporal_Pole_Sup_R, right temporal role: superior temporal gyrus; I: Angular_R, right angular; J: Frontal_Sup_Medial_R, right superior frontal gyurs, medial; K: Supp_Motor_Area_R, right supplementary motor area.

Intergroup differences of results from ALFF analysis were shown in **Table 2**. Compared to HC group, MDD with CM showed increased ALFF in the left orbital part of inferior frontal gyrus (-45, 18, -9. BA47/38), left middle frontal gyrus (-36, 39, 21. BA10/9/46), left medial of superior frontal gyrus (-3, 48, 33. BA9), left supplementary motor area (-3, -3, 75. BA6), left anterior cingulated and paracingulate gyri (-3, 45, 9. BA32), left supramarginal gyrus (-60, -27, 39. BA1/2), left inferior parietal, extending to supramarginal and angular gyri (-45, -48, 57. BA40/7), right orbital part of middle frontal gyrus (48, 51, -6. BA47/10/45), right triangular part of inferior frontal gyrus (51, 33, 18. BA46) and right dorsolateral part of superior frontal gyrus (21, 48, 36. BA9/8) (**Figure 1**). Increased ALFF in MDD without CM, compared to HC group, was observed in left triangular part of inferior frontal gyrus (-48, 48, 3. BA10/46/47), left middle frontal gyrus (-24, 51, 36. BA9), left inferior parietal, extending to supramarginal and angular gyri (-45, -51, 48. BA40/39), left precuneus (-6, -72, 57. BA7), left middle occipital gyrus (-27, -96, 12. BA19), right orbital part of inferior frontal gyrus (54, 42, -6. BA10/47/45), right medial part of superior frontal gyrus (18, 66, 12. BA10/9/6/8), right supplementary motor area (3, -6, 72. BA6), right precuneus (3, -66, 45. BA7), right angular (57, -60, 24. BA39) and right temporal role: superior temporal gyrus (45, 18, -15. BA38/47/34/28) (**Figure 2**).

**Table 2:** The comparison of ALFF in MDD with CM, MDD patients without CM and controls (AlphaSim-corrected, p < 0.01).

Brain areas			BA	Voxels	MNI			*T*-scores	Correlation r&p
Hemisphere	Region	Label			x	y	z		
(*Abuse* > HC)									
Left	Frontal	Inferior frontal gyrus, orbital part	47/38	34	-45	18	-9	4.88	r=-0.06 p=0.81
		Middle frontal gyrus	10/9/46	131	-36	39	21	4.27	r=-0.1 p=0.71
		Superior frontal gyrus, medial	9	16	-3	48	33	3.75	r=-0.09 p=0.74
		Supplementary motor area	6	29	-3	-3	75	3.63	NS
	Cingulate	Anterior cingulated and paracingulate gyri	32	59	-3	45	9	4.76	r=-0.09 p=0.74
	Parietal	Supramarginal gyrus	1/2	20	-60	-27	39	4.87	r=-0.11 p=0.69
		Inferior parietal, extending to supramarginal and angular gyri	40/7	100	-45	-48	57	4.61	r=-0.08 p=0.77
Right	Frontal	Middle frontal gyrus, orbital part	47/10/45	42	48	51	-6	4.57	r=-0.09 p=0.73
		Inferior frontal gyrus, triangular part	46	18	51	33	18	3.39	r=-0.10 p=0.70
		Superior frontal gyrus, dorsolateral	9/8	24	21	48	36	3.66	r=-0.08 p=0.75
(*NS-Abuse* > HC)									
Left	Frontal	Inferior frontal gyrus, triangular part	10/46/47	100	-48	48	3	7.58	NS
		Middle frontal gyrus	9	34	-24	51	36	4.11	NS
	Parietal	Inferior parietal, extending to supramarginal and angular gyri	40/39	217	-45	-51	48	5.70	NS
		Precuneus	7	19	-6	-72	57	3.73	NS
	Occipital	Middle occipital gyrus	19	16	-27	-96	12	4.73	NS
Right	Frontal	Inferior frontal gyrus, orbital part	10/47/45	28	54	42	-6	3.70	NS
		Superior frontal gyrus, medial	10/9/6/8	411	18	66	12	6.35	NS
		Supplementary motor area	6	41	3	-6	72	4.68	NS
	Parietal	Precuneus	7	26	3	-66	45	4.38	NS
		Angular	39	21	57	-60	24	3.75	NS
	Temporal	Temporal pole: superior temporal gyrus	38/47/34/28	51	45	18	-15	4.85	NS
(*Abuse* > *NS-Abuse*)									
Left	Frontal	Middle frontal gyrus	NS	19	-27	48	12	3.79	NS
	Cerebelum		NS	43	-33	-75	-39	4.12	NS
Right	Cerebelum		NS	27	39	-78	-39	4.03	NS

ALFF, amplitude of low frequency fluctuation; MDD, Major Depressive Disorder; Abuse, MMD patients with CM; NS-Abuse, MDD patients without CM; HC, matched healthy controls; MNI, Montreal Neurological Institute; x, y and z, coordinates of primary peak locations in the MNI space.; Correlation, the correlation between ALFF and CM scales.

**Figure 3:**
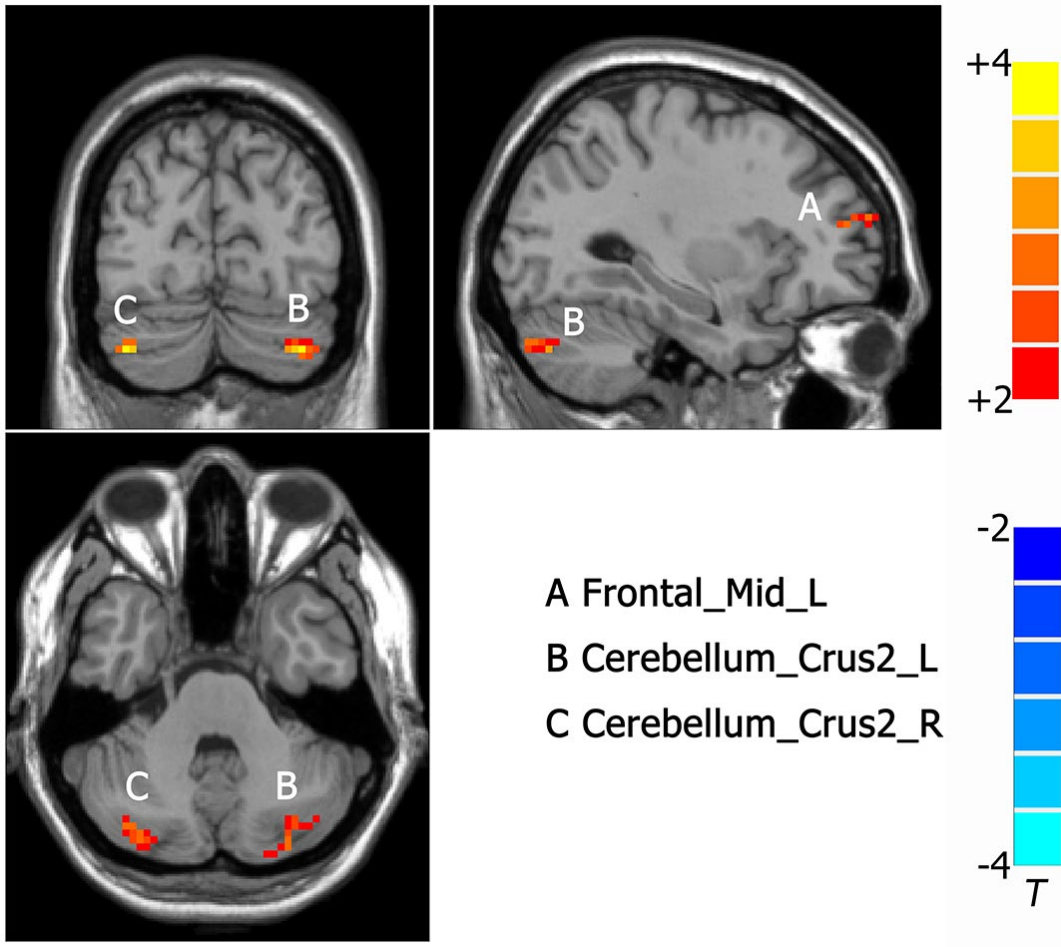
Activated brain regions showed by rs-fMRI using method of ALFF in MDD patients with CM compared with patients without CM. A: Frontal_Mid_L, left middle frontal gyrus; B: Cerebelum_Crus2_L, left cerebellum; C: Cerebelum_Crus2_R, right cerebellum.

Compared to MDD without CM, increased ALFF was observed in the left frontal middle frontal gyrus (-27, 48, 12. NS), left cerebellum (-33, -75, -39. NS) and right cerebellum (39, -78, -39. NS) in MDD with CM (**Figure 3**).

**Figure 4:**
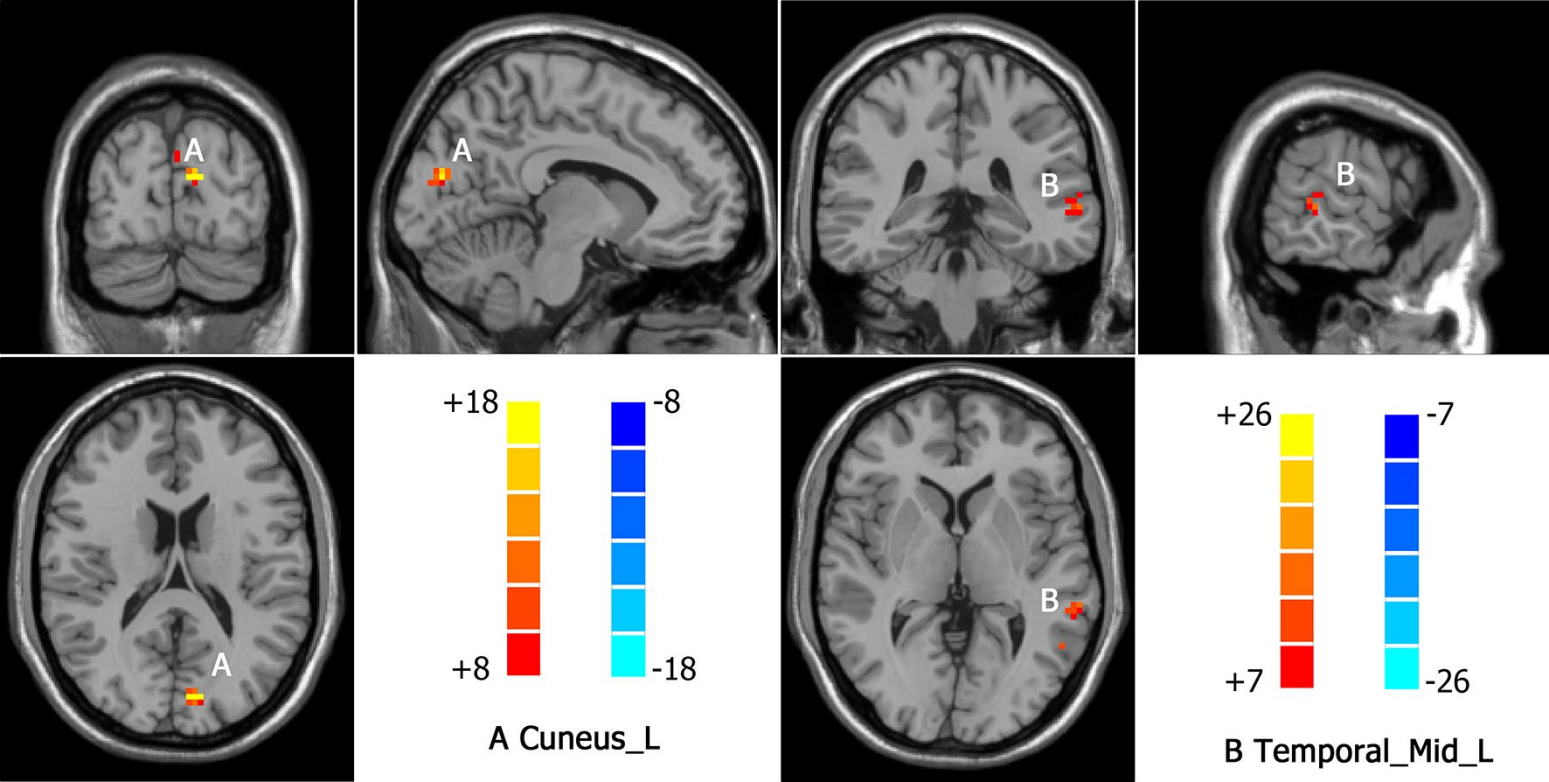
Increased fALFF in MDD patients with CM and patients without CM compared with healthy controls(HC). A: Cuneus_L, left cuneus; B: Temporal_Mid_L, left middle temporal gyrus.

Intergroup differences detected in fALFF analysis were shown in **Table 3**. Compared to HC group, MDD with CM showed increased fALFF in left cuneus (-9, -84, 18. BA19) (**Figure 4**). Increased fALFF in MDD without CM, compared to HC group, was observed in left middle temporal gyrus (-54, -57, 9. BA39) (**Figure 4**).

**Table 3:** The comparison of fALFF in MDD with CM, MDD patients without CM and controls (AlphaSim-corrected, p < 0.01).

Brain areas			BA	Voxels	MNI			*T*-scores	Correlation r&p
Hemisphere	Region	Label			x	y	z		
(*Abuse* > HC)									
Left	Parietal	Cuneus	19	27	-9	-84	18	17.79	r=-0.16 p=0.57
(*NS-Abuse* > HC)									
Left	Temporal	Middle temporal gyrus	39	16	-54	-57	9	26.75	NS
(*Abuse* > *NS-Abuse*)									
NS	NS		NS	NS	NS	NS	NS	NS	NS

fALFF, fractional amplitude of low frequency fluctuation; MDD, Major Depressive Disorder; Abuse, MMD patients with CM; NS-Abuse, MDD patients without CM; HC, matched healthy controls; MNI, Montreal Neurological Institute; x, y and z, coordinates of primary peak locations in the MNI space.; Correlation, the correlation between ALFF and CM scales.

### FC Analysis

Intergroup differences observed in FC analysis were shown in **Table 4**. Left middle frontal gyrus where ALFF was significantly changed between MDD with CM, compared with MDD without CM, was taken as ROI. The left anterior cingulated and paracingulate gyri, left orbital part of inferior frontal gyrus and left inferior parietal, extending to supramarginal and angular gyri where ALFF was altered in MDD with CM and MDD without CM, when compared to HC, were also taken as ROIs.

**Table 4:** The comparison of functional connectivity in MDD with CM, MDD patients without CM to HC (AlphaSim-corrected, p < 0.01).

Regions of interest	Brain areas			BA	Voxels	MNI			*T*-scores	*z*-scores	Correlation r&p
	Hemisphere	Region	Label			x	y	z			
(*Abuse* > HC)											
Left middle frontal gyrus	Left	Frontal	Precentral gyrus	6	19	-45	-6	-60	4.11	0.21	r=-0.09 p=0.72
Left anterior cingulated and paracingulate gyri	Left		Parahippocampal gyrus	36	19	-24	-6	-36	4.95	0.16	r=-0.07 p=0.78
	Right	Frontal	Inferior frontal gyrus, triangular part	NS	25	33	18	27	3.41	0.13	r=-0.23 p=0.40
Left inferior frontal gyrus, orbital part	Left	Frontal	Inferior frontal gyrus, triangular part	NS	21	-24	30	6	3.51	0.21	r=0.23 p=0.39
Left inferior parietal, extending to supramarginal and angular gyri	Left		Fusiform gyrus	19	21	-39	-57	-12	4.273	0.12	r=0.03 p=0.53
(HC > *Abuse*)											
Left middle frontal gyrus	Right	Frontal	Superior frontal gyrus, medial	10	28	6	51	33	3.59	0.05	r=0.11 p=0.67
Left anterior cingulated and paracingulate gyri	Right	Parietal	Superior parietal gyrus	40	28	39	-51	57	3.90	0.45	r=0.15 p=0.59
Left inferior frontal gyrus, orbital part	Left	Frontal	Superior frontal gyrus, medial	10	32	-3	66	0	3.66	0.11	r=0.25 p=0.35
	Right	Frontal	Superior frontal gyrus, medial	8	28	9	42	48	5.01	0.25	r=0.25 p=0.36
Left inferior parietal, extending to supramarginal and angular gyri	Left	Temporal	Inferior temporal gyrus	20	28	-48	-3	-42	4.07	0.07	r=0.06 p=0.82
	Left		Paracentral lobule	6	20	-9	-33	60	3.92	0.14	r=-0.29 p=0.27
	Right	Frontal	Middle frontal gyrus	19	10	45	57	6	3.69	0.05	r=0.68 p<0.01
(*NS-Abuse* > HC)											
Left middle frontal gyrus	Left	Temporal	Inferior temporal gyrus	20	21	-48	-24	-27	3.69	0.20	NS
	Left	Temporal	Middle temporal gyrus	21	28	-60	-39	-6	4.28	0.19	NS
Left inferior parietal, but supramarginal and angular gyri	Right	Temporal	Inferior temporal gyrus	20	20	66	-45	-9	3.29	0.16	NS
(HC > NS-Abuse)											
Left middle frontal gyrus	Left		Fusiform gyrus	19	17	-27	-51	-15	4.26	0.15	NS
	Right		Fusiform gyrus	19	68	24	-48	-15	5.04	0.16	NS
Left inferior parietal, but extending to supramarginal and angular gyri	Left		Precentral gyrus	19	9	-54	0	24	3.84	0.16	NS

MDD, Major Depressive Disorder; Abuse, MMD patients with CM; NS-Abuse, MDD patients without CM; HC, matched healthy controls; MNI, Montreal Neurological Institute; x, y and z, coordinates of primary peak locations in the MNI space. Correlation, the correlation between FC and CM scales.

**Figure 5:**
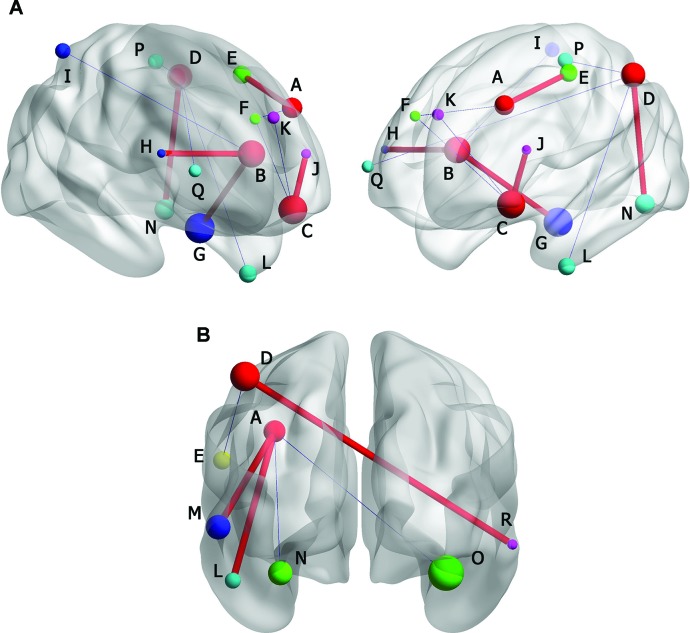
**(A)** Brain functional connectivity (FC) of rs-fMRI in MDD without CM compared with HC. **(B)** FC in MDD patients with CM compared with HC. Size of balls represent the t-scores of every brain regions; the blue thin edge represents the negative FC and the red bold edge represents the positive FC between different brain regions; A: left middle frontal gyrus; B: left anterior cingulated and paracingulate gyri; C: left inferior frontal gyrus, orbital part; D: left inferior parietal, but supramarginal and angular gyri; E: left precentral gyrus; F: right superior frontal gyrus, medial; G: left parphippocampal gyrus; H: right inferior frontal gyrus, triangular part; I: right superior parietal gyrus; J: left inferior frontal gyrus, triangular part; K: left superior frontal gyrus, medial; L: left inferior temporal gyrus; M: left middle temporal gyrus; N: left fusiform gyrus; O: right fusiform gyrus; P: left paracentral lobule; Q: right middle frontal gyrus; R: right inferior temporal gyrus.

In MDD with CM group, positive FC was observed between left middle frontal gyrus and left precentral gyrus (-45, -6, -60. BA6), and negative FC was observed in right medial of superior frontal gyrus (6, 51, 33. BA10). The left anterior cingulated and paracingulate gyri had positive FC with left parahippocampal gyrus (-24, -6, -36. BA36) and right triangular part of inferior frontal gyrus (33, 18, 27. NS). Negative FC was observed in right superior parietal gyrus (39, -51, 57, BA40). Left orbital part of inferior frontal gyrus had positive FC with left triangular part of inferior frontal gyrus (-24, 30, 6. NS) and negative FC was observed in left medial of superior frontal gyrus (-3, 66, 0. BA10) and right medial of superior frontal gyrus (9, 42, 48. BA8). The left inferior parietal, extending to supramarginal and angular gyri, had positive FC with left Fusiform gyrus (-39, -57, -12. BA19). Negative FCs were found in left inferior temporal gyrus (-48, -3, -42. BA20), left paracentral lobule (-9, -33, 60. BA19), and right middle frontal gyrus (45, 57, 6. BA19) (**Figure 5**).

In MDD without CM, positive FC was observed between left middle frontal gyrus and left inferior temporal gyrus (-48, -24, -27. BA20), left middle temporal gyrus (-60, -39, -6. BA21), whereas negative FC was observed in left fusiform gyrus (-27, -51, -15. BA19) and right fusiform gyrus (24, -48, -15. BA19). The left inferior parietal, extending to supramarginal and angular gyri, had positive FC with right inferior temporal gyrus (66, -45, -9. BA20). Negative FC was found in left precentral gyrus (-54, 0, 24. BA19) (**Figure 5**).

### Correlation between Brain Functional Alteration

Pearson Correlation showed that FC alteration between the left inferior, extending to supramarginal and angular gyri, and right middle frontal gyrus, had a positive correlation with CM scale (r = 0.68, p < 0.01). All details are shown in **Table 2**,**3**, **4**.

## Discussion

The current study has investigated the impact of maltreatment during early-life in MDD patients by examining functional activation and connectivity during resting state. To the best of our knowledge, this is the first study that visualizes the whole brain ALFF profiles of MDD with CM during spontaneous brain activity using rs-fMRI. Moreover, FC also has been adopted to detect special brain connectivity. The purpose of this study is to elucidate the mechanism of brain function underlying correlations between MDD and CM experience, based on ALFF and FC results. We also aimed to discover distinct brain regions which could differentiate MDD patients with CM experience from patients without CM.

Firstly, we found that under scan of rs-fMRI, compared with HC, MDD patients with CM had enhanced ALFF in prefrontal-limbic regions, left orbital part of inferior frontal gyrus, right orbital part of middle frontal gyrus, which is similar to the results from previous task-state fMRI studies in MDD with CM (23, 24). In MDD patients without CM, compared with HC, increased ALFF only in the right orbital part of inferior frontal gyrus was found. A depressive patient who had CM experience had more activated OFC than MDD without CM in resting state. Furthermore, brain activity in the right dorsolateral prefrontal cortex (DLPFC) was increased in MDD with CM, but not in MDD without CM. DLPFC had been targeted in transcranial magnetic therapy for MDD. DLPFC is involved in emotional process during the suppression stage, and increased FC was reported in vmPFC (25) and orbitofrontal cortex (OFC) (26). OFC is considered anatomically synonymous with the vmPFC (27–29). The orbitofrontal cortex (OFC) is well-known as a key region for regulating emotion, and any damage in OFC would result in changes in emotion, personality, behavior, and social conduct (30). The loss of volume of OFC was reported in MDD (31, 32). In the present study, both increased OFC and DLPFC were found in MDD with CM. In addition, both precuneus and angular were activated, which play great role in depression (33).

Our results show that ALFF of left anterior cingulated and paracingulate gyri, which belong to the ACC of limbic system, was increased in MDD with CM, whereas no increased ALFF in any subregion of limbic system was detected in MDD without CM. Previous studies indicated that people with CM had a smaller ACC volume than those without CM (34, 35). Task-state fMRI studies demonstrated that vmPFC/ACC activation plays key roles in processing fear, appraising negative emotions and regulating emotional responses *via* the limbic system (36–39). Thus, hyperactivity of ACC may underlie fear dysregulation in MDD with CM, compared with patients without CM. Although abnormal function of amygdale (40) and hippocampus (41) are reportedly associated with MDD and CM, we found no alteration in ALFF in the amygdale and hippocampus. Interestingly, altered ALFF in these brain regions in MDD patients with CM was reported using negative emotional discrimination under task-state fMRI (13, 42, 43). The discrepancy between the results may be explained by the following reasons: for the amygdale, vmPFC have direct white matter fiber projection to the amygdale (44, 45) and have a top-down, inhibitory effect on the amygdale, because OFC and ACC were both found to have increased ALFF in our study, thus the function of amygdale might be inhibited by OFC; as for the hippocampus, relative to the prefrontal cortex (PFC), it matures earlier from perceptive of evolution, thus the hippocampus is less vulnerable to CM experience (23, 46). Moreover, other factors, such as limited sample number, different states (rest instead of task), and different measurements (task activation vs. amplitude), may also affect the difference.

Secondly, our FC study showed that the left inferior frontal gyrus (orbital part) had increased FC with left inferior frontal gyrus (triangular part), and decreased FCs with bilateral superior medial frontal gyrus. Also, the left anterior cingulated, paracingulate gyri had increased FC with left parahippocampal gyrus, and decreased FC with right superior parietal gyrus in MDD with CM, compared with HC, whereas no FC was observed in these ROIs in MDD without CM. Our results also revealed the dysfunction of OFC in MDD with CM, which was consistent with previous FC studies showing increased connection between sub-regions within the orbital and prefrontal cortex (47–49), specific brain areas playing critical roles in MDD’s aberrant networks. Our results showed that the anterior cingulated and paracingulate gyri had decreased FC with superior parietal gyrus, and had increased FC with parahippocampal gyrus, which was similar to results from previous research (50). Given that ACC was a key node in default-mode network and the parahippocampal gyrus essentially involved in memory encoding, aberrant connectivity in MDD with CM may be involved in episodic memory related to experience of CM.

Lastly, after comparing the ALFF between MDD patients with CM and without CM, notably, altered ALFF in the left middle frontal gyrus (LMFG) and cerebellum was unexpectedly detected in MDD with CM compared to those without CM. MFG is a part of frontal lobe which has advanced cognitive function and participates in integrating emotion and information from the internal and external environment, and extracting episodic memory (51, 52). LMFG is located in dorsolateral prefrontal cortex, which has inhibitive (53) and recalling (54) function in psychological disease. LMFG may play a role in extracting unpleasant memory of early-life CM experience, especially memory of disagreeable verbal information, and impaired LMFG function may affect MDD onset. As for cerebellum, its volume declines in patients with MDD (55) and is involved in the modulation of emotional processing and may act as ‘emotional pacemaker’ (56) in MDD. Thus, MDD patients with CM may have a greater increase in brain activity in recalling past sufferings and emotional experience, than patients without CM.

## Conclusion

Our study revealed altered resting-state brain activation in drug-naïve MDD patients with CM experience. The approach using rs-fMRI may be useful to investigate neural mechanisms into how CM affects developmental trajectory of brain maturation, leading to MDD in the later life.

## Limitations

There are two major limitations. Firstly, for Chinese patients with traditional conservative concept in sex, it is difficult to collect any information regarding sex abuse during their childhood. Secondly, CM contains heterogeneous conditions that include emotional abuse, sex abuse, physical abuse, emotional neglect, and physical neglect. We did not separately analyze our results depending on the subtypes of CM in MDD patients due to limited sample number. These limitations will be a line with future inquiry being pursued by our group.

## Ethics Statement

This study was approved by the Ethical Committee at the Third Affiliated Hospital of Beijing University of Chinese Medicine (protocol number: 2015BZHYLL0140). In accordance with the Declaration of Helsinki, all subjects were given written informed consent.

## Author Contributions

ZX, JZ and DW participated in the design of the study, conducted the analyses, and wrote the manuscript. SZ collected the clinical information and performed the HAMD assessment. TW helped with the design and coordination of the study and wrote the manuscript. XR participated in fMRI data collection. XZ and AK contributed to interpretation of the data and drafting the manuscript. MQ and JF conceived and coordinated the design of the study, and wrote the manuscript. All authors read and approved the final manuscript.

## Funding

This work was supported by the National Natural Science Foundation of China (Grant No. 81573905) and Excellent Project of Beijing Municipal Science and Technology Commission (Grant No. Z141107002514080).

## Conflict of Interest Statement

The authors declare that the research was conducted in the absence of any commercial or financial relationships that could be construed as a potential conflict of interest.
